# Quantum dot and electron acceptor nano-heterojunction for photo-induced capacitive charge-transfer

**DOI:** 10.1038/s41598-021-82081-y

**Published:** 2021-01-28

**Authors:** Onuralp Karatum, Guncem Ozgun Eren, Rustamzhon Melikov, Asim Onal, Cleva W. Ow-Yang, Mehmet Sahin, Sedat Nizamoglu

**Affiliations:** 1 Department of Electrical and Electronics Engineering, Koc University, Istanbul, Turkey; 2 Department of Biomedical Sciences and Engineering, Koc University, Istanbul, Turkey; 3 Graduate School of Materials Science and Engineering, Koc University, Istanbul, Turkey; 4 Materials Science and Nano-Engineering Program, Sabanci University, Istanbul, Turkey; 5 Nanotechnology Research and Application Center, Sabanci University, Istanbul, Turkey; 6 Department of Nanotechnology Engineering, Abdullah Gul University, Kayseri, Turkey

**Keywords:** Materials for devices, Quantum dots

## Abstract

Capacitive charge transfer at the electrode/electrolyte interface is a biocompatible mechanism for the stimulation of neurons. Although quantum dots showed their potential for photostimulation device architectures, dominant photoelectrochemical charge transfer combined with heavy-metal content in such architectures hinders their safe use. In this study, we demonstrate heavy-metal-free quantum dot-based nano-heterojunction devices that generate capacitive photoresponse. For that, we formed a novel form of nano-heterojunctions using type-II InP/ZnO/ZnS core/shell/shell quantum dot as the donor and a fullerene derivative of PCBM as the electron acceptor. The reduced electron–hole wavefunction overlap of 0.52 due to type-II band alignment of the quantum dot and the passivation of the trap states indicated by the high photoluminescence quantum yield of 70% led to the domination of photoinduced capacitive charge transfer at an optimum donor–acceptor ratio. This study paves the way toward safe and efficient nanoengineered quantum dot-based next-generation photostimulation devices.

## Introduction

Neural interfaces that can supply electrical current to the cells and tissues play a central role in the understanding of the nervous system. Proper design and engineering of such biointerfaces enables the extracellular modulation of the neural activity, which leads to possible treatments of neurological diseases like retinal degeneration, hearing loss, diabetes, Parkinson and Alzheimer^1–3^. Light-activated interfaces provide a wireless and non-genetic way to modulate neurons with high spatiotemporal resolution, which make them a promising alternative to wired and surgically more invasive electrical stimulation electrodes^4,5^.

The charge injection mechanism at the device/tissue interface is an important parameter that affects the efficiency and safety of neuromodulating devices. The safe modulation of neural activity requires the avoidance of irreversible faradaic reactions, which might be harmful to the biological tissues^6,7^. In that regard, capacitive stimulation is accepted as a safe method that modulates the cell membrane by inducing transient displacement currents without any direct charge transfer from the electrode to the biological medium^6,7^. Hence, both electrical and optical neurostimulators are material- and device-wise engineered to induce capacitive currents. For example, titanium nitride (TiN)-based capacitive electrodes for electrical stimulation inject charges through the electrode–electrolyte double layer^8^. The capacity of such electrodes charging and discharging the double layer can be improved by additional dielectric coatings of tantalum/tantalum oxide (Ta/Ta_2_O_5_). Moreover, silicon, organic semiconductors and carbon nanotubes have been used for capacitive photostimulation of neurons^9–13^.

Colloidal quantum dots (QDs) are promising nanomaterials for neural interfaces due to their advantageous structural and optoelectronic properties such as tunable bandgap, high absorption in the visible spectrum, solution processability and stability^14^. Photostimulation devices based on HgTe, CdSe and InP QDs were previously reported in the literature that can efficiently stimulate neurons and evoke action potentials^15–18^. These devices, however, either contain toxic-heavy-metals or operate photoelectrochemically, both of which might harm the tissues in the long-term use. Heavy-metal-free QD-based neural interfaces that have dominant capacitive charge injection have not been reported in the literature yet.

In this study, we demonstrate QD-fullerene donor–acceptor nano-heterojunction photoelectrodes that produce capacitive-dominant photoresponse. For that we nanoengineer toxic-heavy-metal-free InP-based QDs and QD-fullerene nano-heterojunctions. While InP/ZnS QD leads to faradaic charge transfer, lower exciton binding energy and better passivation of surface traps of InP/ZnO/ZnS nanostructure facilitated capacitive charge transfer, which is maximized by tuning the donor–acceptor ratio. Therefore, we found out that the carrier localization and surface states of quantum dots at the nano-heterojunction has a vital role for the control of the bioelectrical currents.

## Results

**Figure 1:**
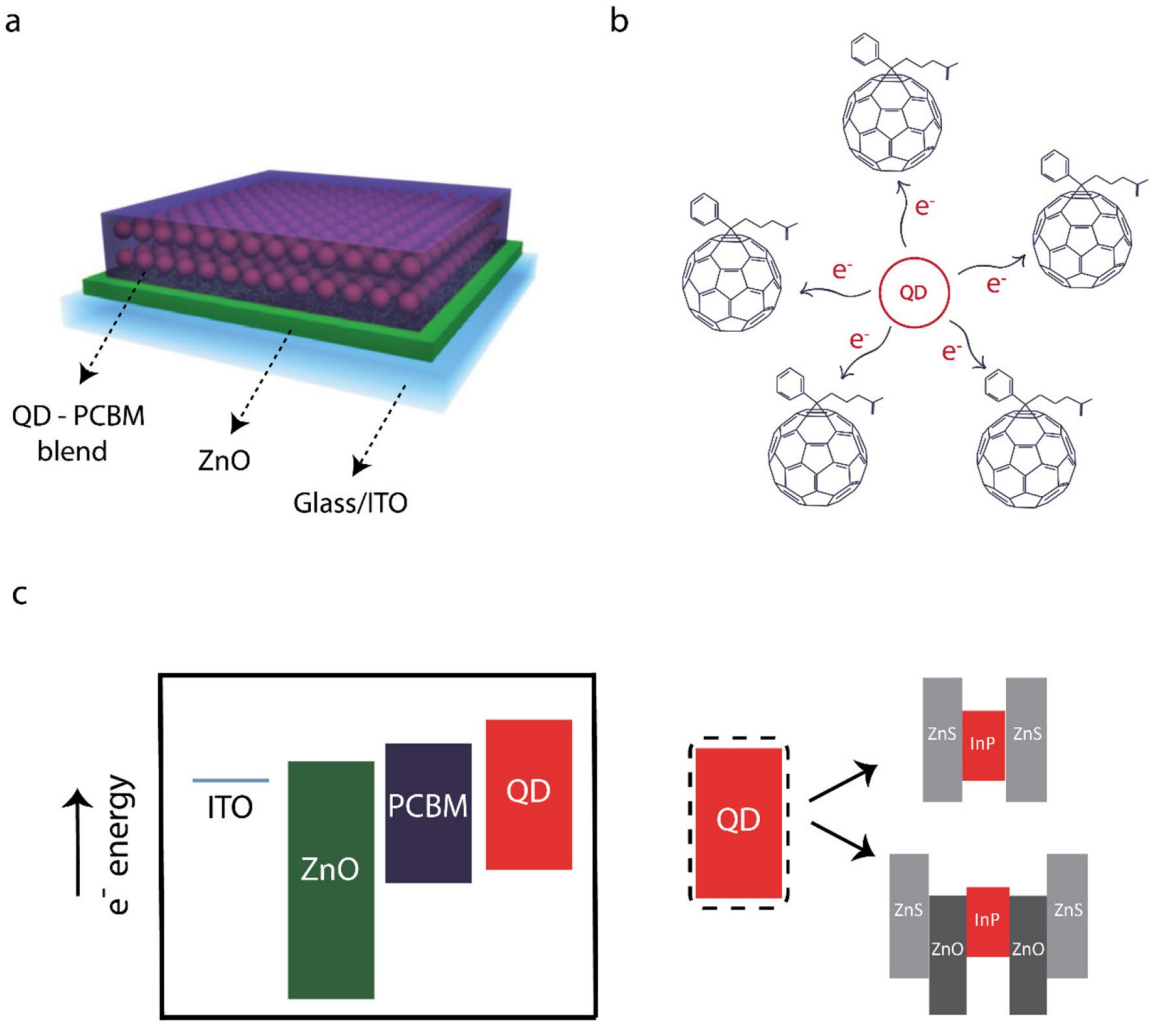
(**a**) The schematic of the quantum dot and PCBM nano-heterojunction device structure, and (**b**) the electron transfer from the InP-based quantum dots to PCBM. (**c**) The energy band alignment based on our previous reports^20,21^. InP/ZnS core/shell and InP/ZnO/ZnS core/shell/shell QDs were incorporated into the photoelectrode architecture.

Figure 1 demonstrates the device architecture and corresponding energy band diagram of the photoelectrodes. The intermediate layer of ZnO nanoparticles serves for electron transport and hole blocking purposes due to its high electron mobility and large energy barrier at the valence band, respectively. Owing to the electron accepting property of fullerenes and the energy alignment of the QD and [6,6]-phenyl C61 butyric acid methylester (PCBM), the photoactive layer consisting of InP-based QD and a fullerene derivative of PCBM forms a donor–acceptor nano-heterojunction. PCBM captures the photogenerated electrons from the quantum dots and the holes remain in the QDs^19^.

**Figure 2:**
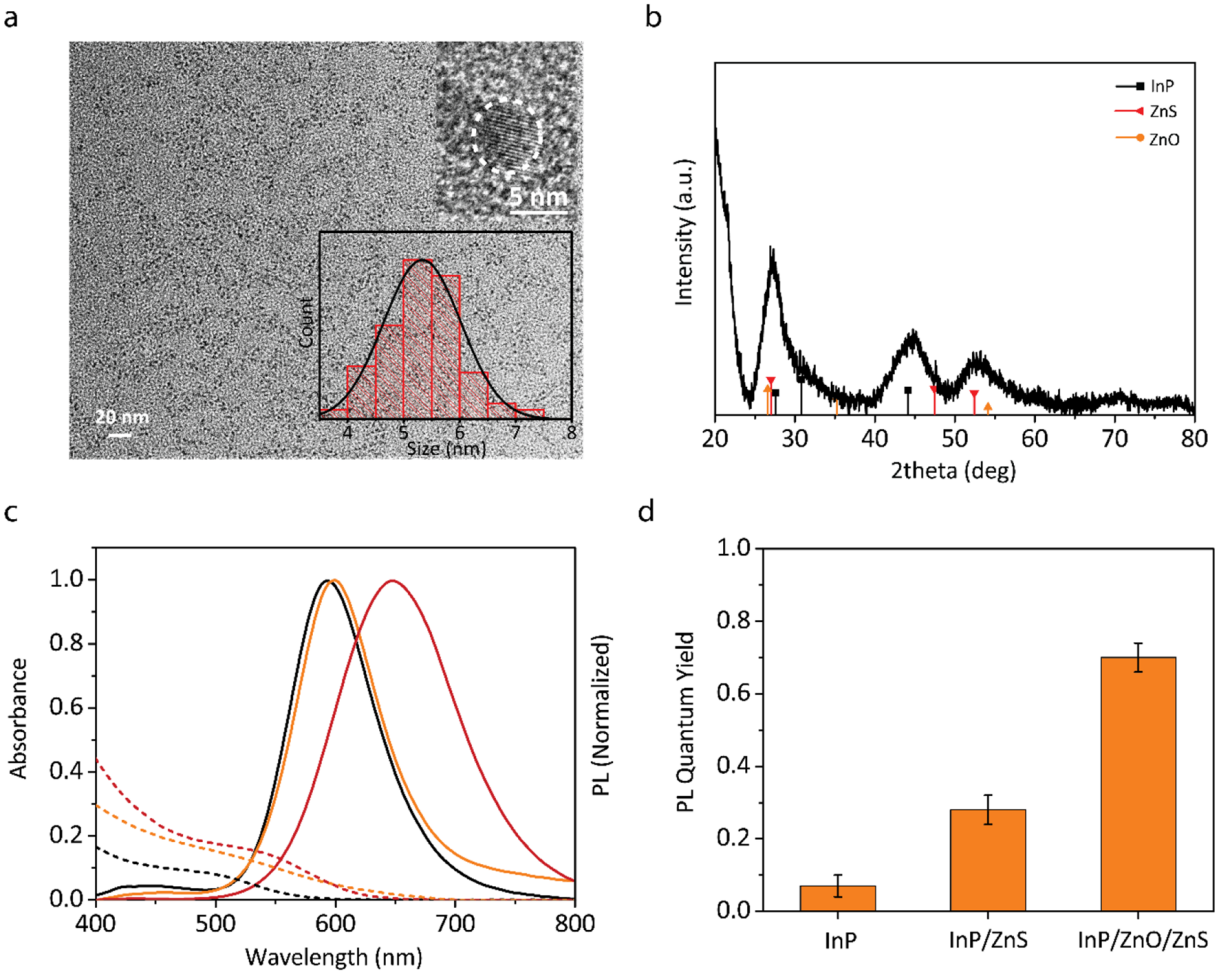
Structural and optical properties of QDs. (**a**) TEM image of InP/ZnO/ZnS QD. Scale bar is 20 nm. Insets: HR-TEM image of InP/ZnO/ZnS QD with 5 nm scale bar (top), the size distribution (N = 200) (bottom). (**b**) XRD pattern of InP/ZnO/ZnS QD. (**c**) Absorption and emission spectrum of InP core (black), InP/ZnS (orange) and InP/ZnO/ZnS (red) QDs dispersed in toluene each at the concentration of ~ 0.5 micromolar. (**d**) PL quantum yields of InP, InP/ZnS and InP/ZnO/ZnS QDs (measured in an integrating sphere with excitation wavelength of 375 nm).

InP-based QDs were used for bio-applications because of their intrinsically non-toxic and heavy metal free composition^22^. We synthesized InP core QDs via hot injection method and grew ZnS and ZnO shells for the formation of InP/ZnS core/shell and InP/ZnO/ZnS core/shell/shell nanostructures (see “Methods” for the details of the synthesis)^17,23^. The transmission electron microscopy (TEM) analyses of InP core, InP/ZnS, InP/ZnO and InP/ZnO/ZnS QDs reveal their mean particle sizes as 3.3 nm, 4.7 nm, 4.3 nm and 5.3 nm, respectively, indicating revealing the successful shell growth. (see Fig. 2a and the supporting information). X-ray diffraction (XRD) analysis of InP/ZnO/ZnS QDs shows the corresponding crystallographic diffraction peaks of InP, ZnS and ZnO, indicating the formation of InP/ZnO/ZnS core/shell/shell nanostructures (Fig. 2b) X-ray photoelectron electroscopy (XPS) analysis of InP core, InP/ZnO, InP/ZnO/ZnS and energy dispersive X-ray spectroscopy (EDS) of InP/ZnO/ZnS were provided in Supporting Information to further confirm the growth of ZnO and ZnS shells in InP/ZnO/ZnS core/shell/shell nanostructure.

We next investigated the optical properties of the QDs. Figure 2c shows the absorption and photoluminescence (PL) spectrum of InP core, InP/ZnS and InP/ZnO/ZnS QDs. The conduction band energy levels of InP and ZnO were expected to lead to a type-II behavior in InP/ZnO/ZnS QD, where electron tends to delocalize to the ZnO shell, while the hole is confined in the core. This causes a significant red-shift in the absorption and emission spectrum of InP/ZnO/ZnS compared to InP core and type-I InP/ZnS QDs (Fig. 2c) (normalized absorption profiles can be seen in Supplementary Fig. S4). Moreover, growing ZnS and ZnO shells on InP core leads to enhanced photoluminescence quantum yield (PLQY) values, which are 7%, 28%, and 70% for InP core, InP/ZnS, and InP/ZnO/ZnS, respectively (Fig. 2d). The significant enhancement in the PLQY of InP/ZnS and InP/ZnO/ZnS nanocrystals indicates the successful passivation of nonradiative surface states^24^.

**Figure 3:**
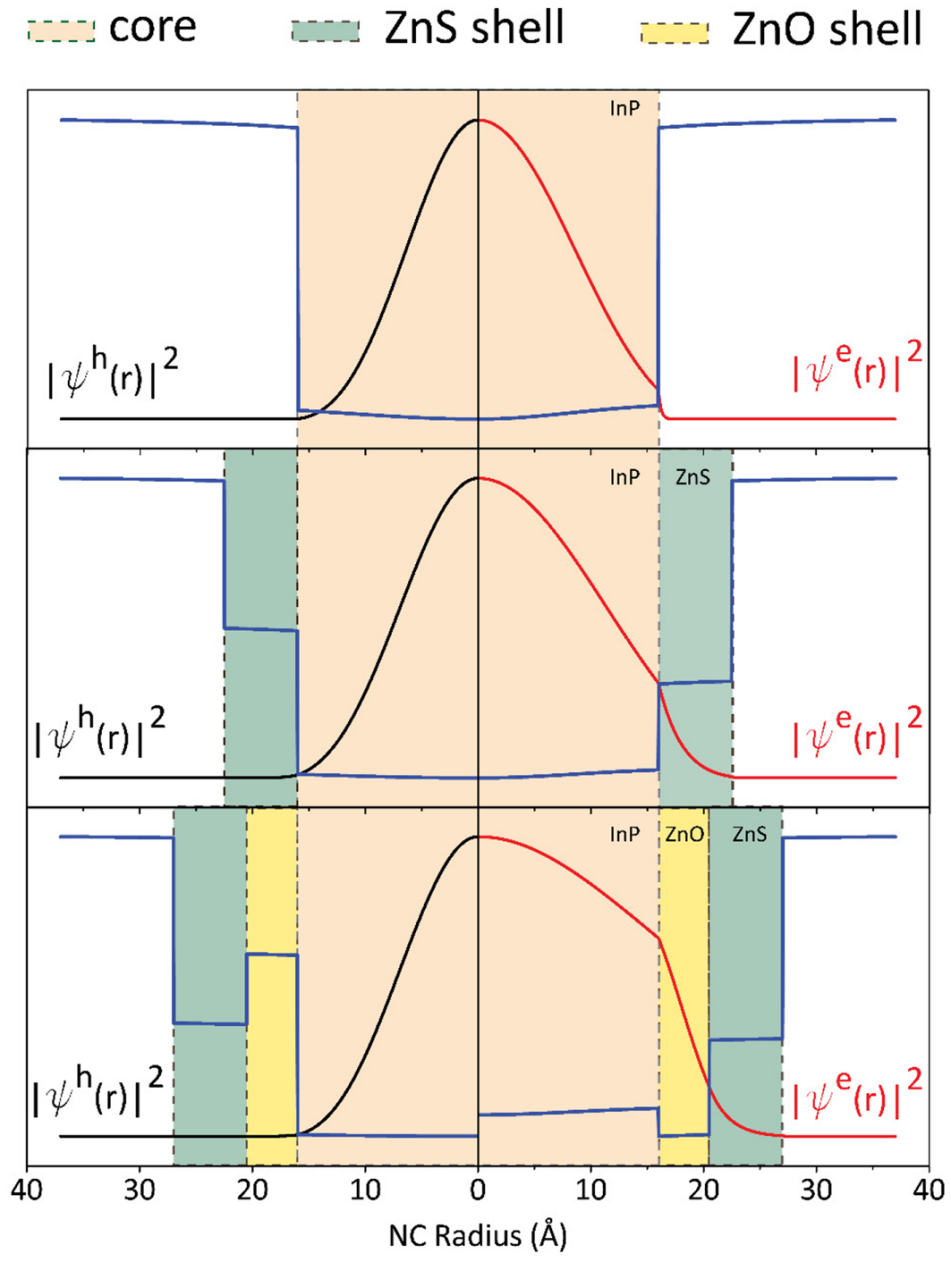
Radial distributions of electrons and holes throughout the InP core (top panel), InP/ZnS core/shell (middle panel) and InP/ZnO/ZnS core/shell/shell (bottom panel) quantum dots. The colored areas show the core region, ZnS shell region, and ZnO shell region. Blue lines show the respective conduction and valence band potential profile of each QD. The bending of the potential profiles is due to attractive Coulomb potential between the electron and hole.

To investigate the electronic properties of the QDs in detail, we conducted quantum mechanical calculations by solving self-consistently the Poisson–Schrödinger equations in the effective mass approximation and using BenDaniel–Duke boundary conditions^29^. The material parameters used in the calculations are listed in Table 1. All Coulombic interactions have been taken into account on both energy eigenvalues and wavefunctions^29^. At the end of the calculations, single particle energies of electron and hole and corresponding radial wavefunctions have been determined. Using these values, exciton binding energies, overlap integrals, oscillator strengths, absorption wavelengths, and transition energies have also been calculated. Figure 3 shows the confinement potential profiles, and electron and hole density functions for InP core (top panel), InP/ZnS core/shell (middle panel) and InP/ZnO/ZnS core/shell/shell (bottom panel) QDs. It should be noted that the maximum values of the density functions are normalized to unity for the consistency of scaling. When we look at the top panel of the Fig. 3, we see that both electron and hole wavefunctions are confined completely in the core region meaning that the exciton binding energy will be large due to strong attractive Coulomb interaction between the electron and hole. In InP/ZnS core/shell QD, although the electron and the hole are still confined to the InP core, a small portion of the electron wavefunction penetrates to the ZnS shell and hence, it is expected that the exciton binding energy will be smaller when it is compared to the single InP core QD. The exciton binding energies in these structures is calculated as 116 meV and 100 meV for InP and InP/ZnS QD, respectively. Moreover, although InP/ZnO/ZnS QD has a type-II energy band alignment as seen from potential profile in the bottom panel of Fig. 3, the electron density does not localize completely in ZnO shell and expands through the whole structure due to its light effective mass, smaller spatial volume and also smaller potential depth, while the hole is completely confined in the InP core. This spatial expansion of electron density decreases the attractive Coulomb energy, i.e. the binding energy, between electron and hole. As a result, InP/ZnO/ZnS QD has a lower binding energy, i.e. 88 meV, compared to the binding energies of type-I InP core and InP/ZnS core/shell QDs. Electron delocalization to the shell indicates a possible transition from type-I to type-II heterostructure^17^, which are known to be more favorable for photovoltaic applications and exciton dissociation^30,31^. This transition can also be deduced from the electron–hole wavefunction overlap ratio. The overlap value is 0.89 for InP core QD and 0.76 for InP/ZnS core/shell QD, while it is considerably lower (0.52) for InP/ZnO/ZnS QD.

**Table 1:** Materials parameters used in the calculations.

Materials	m_e_/m_0_	m_h_/m_0_	ε_s_/ε_0_	E_c_ (eV)	E_v_ (eV)	E_g_ (eV)	E_p_ (eV)
InP	0.08^25^	0.69^25^	12.9^25^	− 4.8^26^	− 6.14^26^	1.34^26^	17^25^
ZnS	0.25^25^	0.59^25^	8.9^25^	− 3.9^26^	− 7.62^26^	3.72^26^	
ZnO	0.24^27^	0.78^27^	8.8^28^	− 5.18^26^	− 8.58^26^	3.40^26^

**Figure 4:**
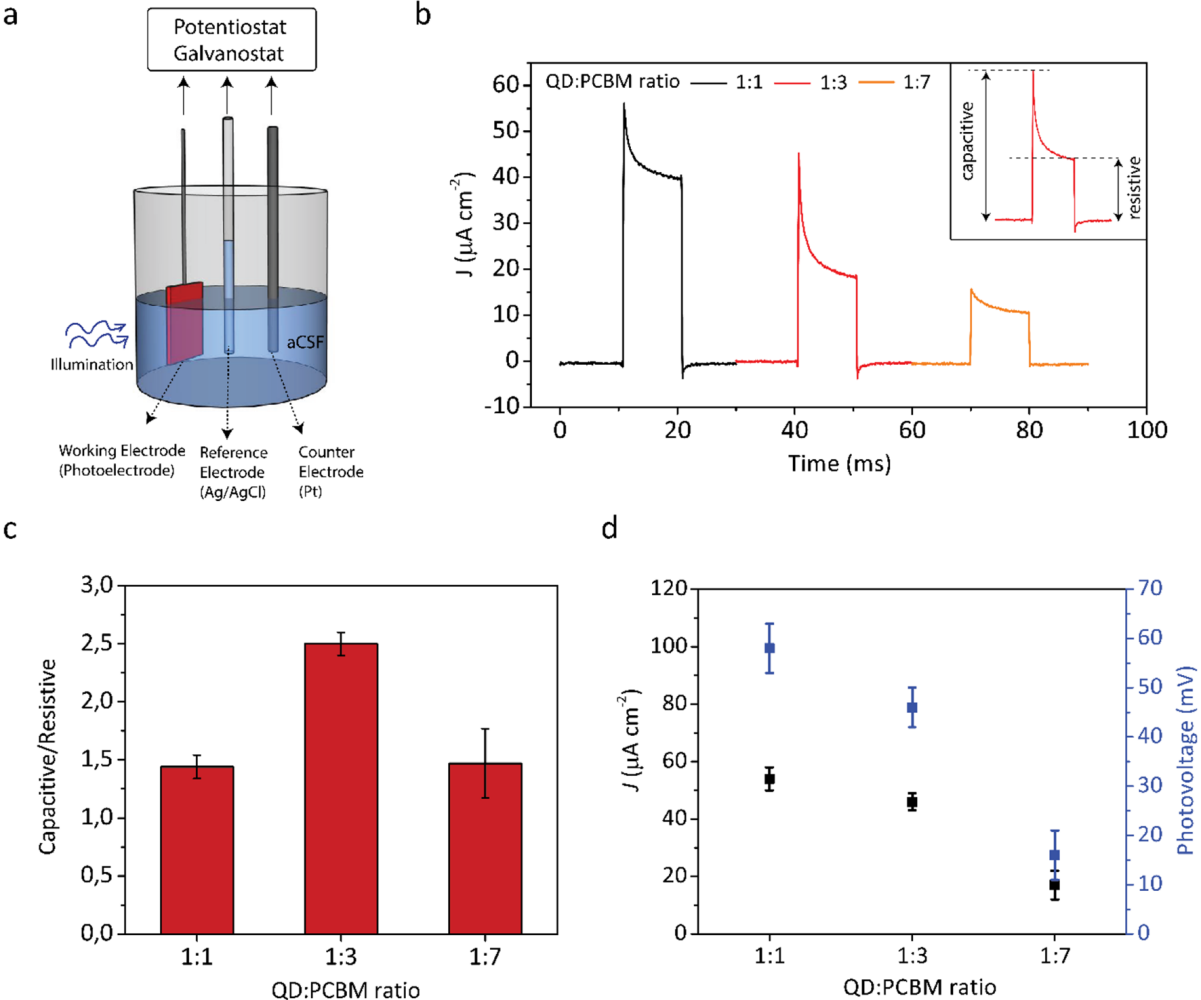
Photocurrent measurement configuration and response of InP QD-based devices. (**a**) Three-electrode photocurrent/photovoltage measurement setup used for the photoresponse analysis of the photoelectrodes. (**b**) Photocurrent density traces of the devices with InP/ZnO/ZnS:PCBM volume ratios of 1:1 (black), 1:3 (red), 1:7 (orange). Inset shows the components of the photocurrent. Capacitive and resistive components of the photocurrents were defined based on another study^9^. Capacitive current is the peak photocurrent reached after the light onset, while resistive current is the photocurrent remained after 90% of the illumination duration passed. (**c**) The ratios of the capacitive to resistive components for devices with different QD:PCBM mixing ratios. (**d**) Peak photocurrent density and photovoltage values for different mixing ratios. Illumination: 10 ms pulsed LED with 445 nm nominal wavelength and optical power density of 57 mW cm^−2^ (N = 4).

We investigate the photoresponse of InP/ZnO/ZnS QD-PCBM nano-heterojunction photoelectrodes (see Supplementary information for the preparation of QD:PCBM blend and the corresponding absorption profiles in Supplementary Fig. S5). Figure 4a shows the photocurrent/photovoltage measurement configuration. We use a three-electrode electrochemical setup while illuminating the samples with a pulsed LED (see “Methods” for the details of the measurement setup). The photoresponse analysis was performed in the artificial cerebrospinal fluid (aCSF) to mimic the working conditions in a biological medium (see “Methods” for the preparation of the aCSF). InP/ZnO/ZnS QD-based devices display rapid charging/discharging spikes with rise/fall times of 200 µs (Fig. 4b). The amplitude of the transient capacitive peaks for the QD:PCBM 1:1 and QD:PCBM 1:3 devices (57 µA cm^−2^ and 45 µA cm^−2^) are close to each other, while it is substantially lower for the QD:PCBM 1:7 devices (15 µA cm^−2^) (Fig. 4b) (1:1, 1:3, 1:7 are the QD:PCBM volume ratios in the blend). Here, QD-PCBM blend consists of electron-donating QD and electron-accepting PCBM, and the number of acceptors per each donor, i.e. the QD:PCBM ratio, affect the photoresponse of the device^31^. Sufficiently high number of acceptors per each donor lead to an efficient charge separation. However, at QD:PCBM 1:7 ratio, the imbalance of acceptors per each donor prevents the effective transfer of separated charges to the other layers due to unbalanced charge transportation between QD and PCBM materials^32^. Moreover, the ratio of capacitive current to electrochemical current is maximum in the QD:PCBM 1:3 device (2.5), while it is similar for QD:PCBM 1:1 and QD:PCBM 1:7 devices (1.43 and 1.47) (Fig. 4c). This indicates that the most effective photoactive layer for the highest ratio of capacitive photocurrent is InP/ZnO/ZnS-PCBM 1:3 blend, which provides both high transient peak and 2.5 times greater capacitive photocurrent compared to the photoelectrochemical/resistive current (Fig. 4c). The corresponding photovoltage values for the devices with different ratios of QD:PCBM are shown in Fig. 4d. These photovoltages show the highest values that can be supplied by the devices. Our best performing QD:PCBM 1:3 device can generate 46 ± 4 mV photovoltage, which would be sufficient to induce action potentials on excitable cells^33^.

**Figure 5:**
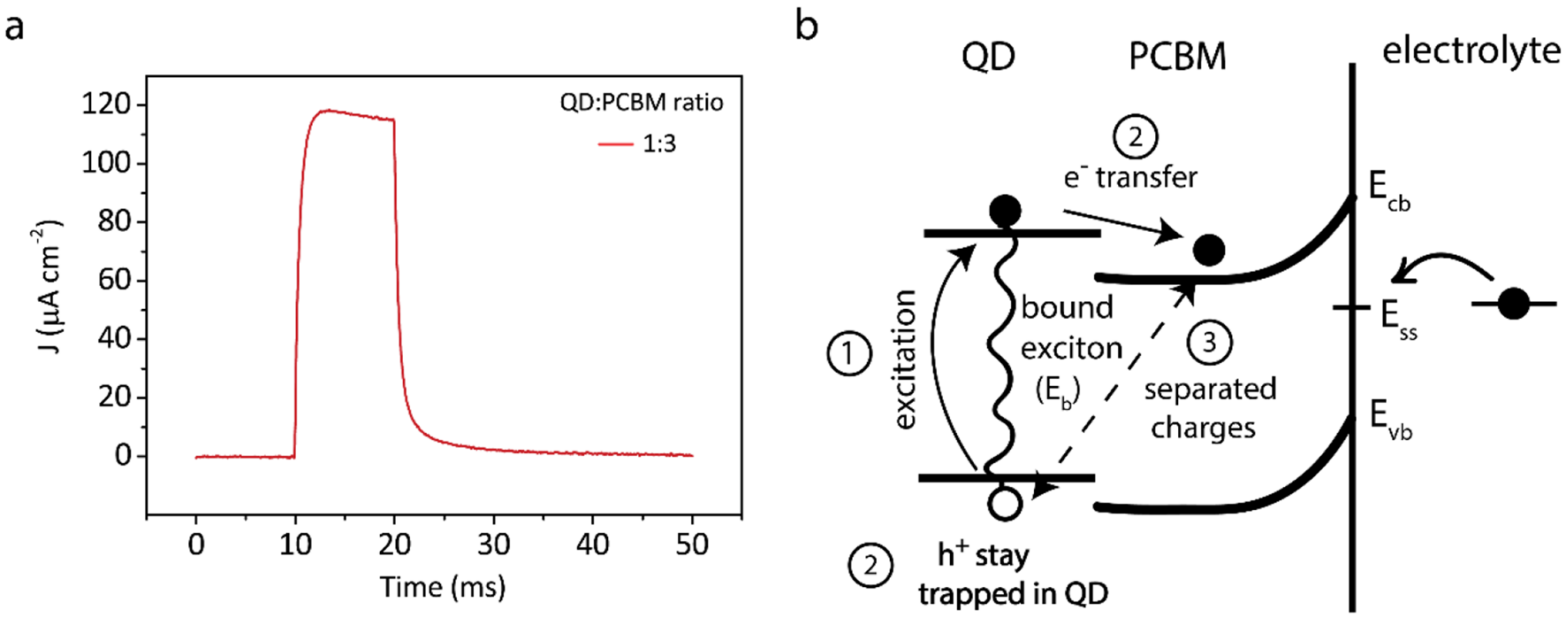
(**a**) Photocurrent density of the InP/ZnS-based photoelectrode with InP/ZnS:PCBM ratio of 1:3. (**b**) Schematic showing qualitatively the proposed ongoing processes at the QD:PCBM-electrolyte interface upon illumination. The processes are numbered according to their occurrence sequence. E_cb_, E_vb_, E_ss_ stands for conduction band, valence band and surface state energy levels, respectively.

Figure 5a shows the photoelectrical response of the nano-heterojunction devices with InP/ZnS quantum dot at the QD:PCBM 1:3 ratio. The steady-state photocurrent under continuous light intensity indicates that the photocurrent is dominated by electrochemical processes. The trace in Fig. 5a also shows that the InP/ZnS-based device shows significantly slower charging/discharging dynamics with rise/fall times of 2 ms compared to 200 µs of InP/ZnO/ZnS based devices. The reasons behind the different behaviors of InP/ZnS-based and InP/ZnO/ZnS-based devices can be attributed to the processes at the QD-PCBM nano-heterojunction and electrolyte interface (Fig. 5b). The incident light pulse will be absorbed by quantum dots, leading to the generation of electron–hole pairs. These electron–hole pairs are bound together with an exciton binding energy, E_b_, which were calculated for each QD via the quantum mechanical simulations. For efficient charge separation, the energy offset between the lowest unoccupied molecular orbit (LUMO) levels of the donor and acceptor materials should be sufficiently large to overcome E_b_^34^. Our quantum mechanical simulations showed that type-II InP/ZnO/ZnS QD has a significantly lower E_b_ compared to InP/ZnS. Hence, InP/ZnO/ZnS-based devices have a more efficient charge separation compared to InP/ZnS-based ones. Besides, even though excitons are formed also in PCBM, those excitons are not expected to reach the donor–acceptor interface due to the small exciton diffusion length in PCBM^35,36^. Thus, the excitonic contribution of PCBM to the photocurrent is almost negligible (see Supplementary Fig. S11 for photocurrent of the devices without QDs).

At the QD-PCBM interface, the electron is transferred to PCBM, while the hole stays trapped in the QD. Due to the much higher molar ratio of PCBM in the QD:PCBM blend, each QD is surrounded by multiple PCBM (check the schematic in Fig. 1b for a qualitative demonstration). Thus, most of the device-electrolyte interface will be covered by PCBM. The potential barrier due to the band bending at the PCBM-electrolyte interface blocks the electrons from migrating to the solution and ZnO layer captures the electrons. As a result, holes trapped in the QDs, which are away from the surface, induce the formation of an oppositely charged double layer at the electrolyte interface, generating a capacitive photocurrent. The direction of the photocurrent from the electrode to electrolyte also confirms the hole-based displacement current. However, the existence of the surface states still mediate charge exchange at the interface, leading to photoelectrochemical processes^37^. Owing to its higher PL QY and core/shell/shell nanostructure of InP/ZnO/ZnS, which implies an improved passivation of surface states compared to InP/ZnS, the InP/ZnO/ZnS-based interfaces advantageously have low photoelectrochemical charge transfer with the electrolyte. Consequently, while type-I InP/ZnS-based devices produce predominantly photoelectrochemical current, type-II InP/ZnO/ZnS-based devices can generate capacitive currents, which was facilitated by the effective charge separation and less amount of nonradiative recombination sites of InP/ZnO/ZnS QDs.

## Conclusion

In this study, we report the successful demonstration of heavy-metal-free and capacitive QD:fullerene nano-heterojunction devices for prospective non-invasive QD-based neurostimulation applications. For that, we synthesized type-I InP/ZnS core/shell and type-II InP/ZnO/ZnS core/shell/shell nanostructures. We tested their photoresponses in a nano-heterojunction of QD:PCBM donor–acceptor system for effective exciton dissociation and charge transfer. InP/ZnO/ZnS-based nano-heterojunction achieved photocurrent with capacitive dominance, which has 2.5 times higher capacitive photocurrent than the resistive photocurrent, while InP/ZnS-based devices are predominantly governed by photoelectrochemical processes. We ascribe that difference to the following two main reasons: (i) The type-II band structure of InP/ZnO/ZnS QD results in a lower exciton binding energy compared to type-I InP/ZnS, leading to more effective charge separation. (ii) The core/shell/shell composition of InP/ZnO/ZnS yields fewer surface states, which diminishes the possible charge transfer between electrolyte and surface states. The conceptual understanding and the unconventional device structures in this study pave the way toward safe and effective quantum dot-based biointerfaces.

## Methods

### InP core, InP/ZnS core/shell, InP/ZnO/ZnS core/shell/shell QD synthesis

For InP core synthesis, we mixed 0.01 mmol stearic acid, 0.01 mmol zinc undecylenate and 0.2 mmol hexadecylamine in 6 mL 1-Octadecene in a three-neck flask. Then, 0.1 mmol indium chloride was injected into the flask in inert atmosphere. The temperature of the solution was increased to 120 °C. To obtain water-free and oxygen-free reaction environment, 20 min evacuation was applied. We then apply refilling in the nitrogen atmosphere and increase the temperature to 230 °C. After the solution reaches 230 °C, we inject 0.2 mmol stock solution of Tris(trimethylsilyl) phosphine into the reaction. The solution was kept at 230 °C 20 min. We cool down the solution to the room temperature and allocate half of it, labeling it as InP Core.

For ZnS shelling, we add 0.15 mmol zinc diethyldithiocarbamate and 2 mL 1-Octadecene into the remaining InP core solution. We increase the reaction temperature to 180 °C and leave at that temperature 30 min under rigorous stirring. Then, we cool down the solution to room temperature. We wash the solution with toluene and ethanol. Finally, we re-disperse the purified solution in toluene.

For ZnO shelling, first, the InP core solution was cooled down to 80 °C. ZnO stock solution was prepared by adding 245 µL oleylamine (OAM), 8 µL oleic acid (OA) and 6,5 mg zinc acetylacetonate hydrate into 1.6 mL 1-octadecene. Afterwards, ZnO stock solution was heated to 80 °C, and mixed until zinc acetylacetonate hydrate was completely dissolved. Then, the ZnO stock solution was added to InP QD solution at 80 °C. Then the solution was heated to 250 °C and kept stirring at that temperature for 30 min. Then, we cool down the solution to room temperature. We wash the solution with toluene and ethanol. Finally, we re-disperse the purified solution in toluene.

### Photoelectrode fabrication

To clean the samples before the fabrication, the unpatterned Glass/ITO substrates were sonicated in Hellmanex III solution (1.5% in deionized water), deionized water, acetone, and IPA consecutively for 15 min each. After drying the samples under 50 °C for 20 min, the substrates went through UV ozone treatment for 15 min. 0.45 M ZnO precursor sol–gel solution was prepared by mixing 219.3 mg zinc acetate dehydrate (Zn(CH_3_CO_2_)_2_·2H_2_O), and 73 mg of Ethanolamine (HOCH_2_CH_2_NH_2_) in 2 mL 2-Methoxyethanol (C_3_H_8_O_2_). The mixture was ultrasonicated at 50 °C for 15 min to obtain a uniform solution. The ZnO precursor sol–gel solution was spin coated on cleaned substrates at 2000 rpm for 60 s, followed by baking at 290 °C for 15 min. For the active layer, InP/ZnS:PCBM and InP/ZnO/ZnS:PCBM solutions were prepared by mixing previously prepared QD solutions in toluene (~ 30 M$\upmu$) and PCBM solution in o-dichlorobenzene (30 mg mL^−1^) with a volume ratios of QD:PCBM 1:1, 1:3, 1:7. The mixture was stirred for 1 h to obtain uniform QD:PCBM solutions. The QD:PCBM layer was formed by spin coating 50 μL of the QD:PCBM solution at 2000 rpm for 60 s, followed by baking at 120 °C for 15 min.

### Optical characterization

Edinburgh Instruments Spectrofluorometer FS5 was used to characterize the optical properties (UV/vis absorption, photoluminescence, quantum yield) of QDs. Integrating sphere module was used for the quantum yield measurements.

### Photocurrent measurements

The photoresponses of the devices were measured in three-electrode electrochemical measurement with Ag/AgCl as the reference electrode, platinum rod as the counter electrode and the thin film samples as the working electrode in aCSF solution by dipping 1 cm^2^ active area of each sample using Autolab Potentiostat Galvanostat PGSTAT302N (Metrohm, Netherlands). aCSF solution was prepared using the following materials stirred in deionized water: 10 mM of 4-(2-hydroxyethyl)-1-piperazineethanesulfonic acid (HEPES), 10 mM of glucose, 2 mM CaCl_2_, 140 mM of NaCl, 1 mM of MgCl_2_, 3 mM of KCl. After obtaining uniform solution, NaOH was slowly added until the pH of aCSF solution reaches 7.4. For illumination, 10 ms light pulses (Thorlabs M450LP1 LED, 445 nm peak wavelength) were applied. Thorlabs DC2200 Driver was used to control the LED. Optical power was measured via Newport 843-R power meter.

### X-ray photoelectron spectroscopy (XPS)

The XPS spectra of quantum dot samples were taken by a Thermo Scientific K-Alpha spectrometer using an Aluminum anode (Al Kα = 1468.3 eV) at an electron take-off angle of 90° (between the sample surface and the axis of the analyzer lens). The spectra were recorded using an Avantage 5.9 data system. The binding energy scale was calibrated by assigning the C1 s signal at 284.5 eV.

### Energy dispersive X-ray spectroscopy (EDS)

EDS was performed using a JEOL Centurio detector, with a spot size of *ca.* 1 Å and probe current of 700 pA. Analysis was conducted on a JEOL JEM-ARM200CF spherical aberration-corrected scanning transmission electron microscope (STEM) operated with an accelerating voltage of 200 keV.

## Supplementary Information

Supplementary information.
