# Supplementary material for: Quantum dot and electron acceptor nano-heterojunction for photo-induced capacitive charge-transfer
Source: Sci Rep. 2021 Jan 28;11:2460. doi: 10.1038/s41598-021-82081-y (PMC7843732; doi:10.1038/s41598-021-82081-y)
Supplement: Supplementary file 1 — Supplementary information. [file 41598_2021_82081_MOESM1_ESM.docx]

Supporting Information

**Quantum Dot and Electron Acceptor Nano-Heterojunction**

**for Photo-induced Capacitive Charge-Transfer**

Onuralp Karatum^1^, Guncem Ozgun Eren^2^, Rustamzhon Melikov^1^, Asim Onal^3^, Cleva W. Ow-Yang^4,5^, Mehmet Sahin^6^, Sedat Nizamoglu^1, 2, 3, *^

^1^ Department of Electrical and Electronics Engineering, Koc University, Istanbul, Turkey

^2^ Department of Biomedical Science and Engineering, Koc University, Istanbul, Turkey

^3^ Graduate School of Materials Science and Engineering, Koc University, Istanbul, Turkey

^4^ Materials Science and Nano-Engineering Program, Sabanci University, Istanbul, Turkey

^5^ Nanotechnology Research and Application Center, Sabanci University Istanbul, Turkey

^6^ Department of Nanotechnology Engineering, Abdullah Gul University, Kayseri, Turkey

* Corresponding Author: snizamoglu@ku.edu.tr


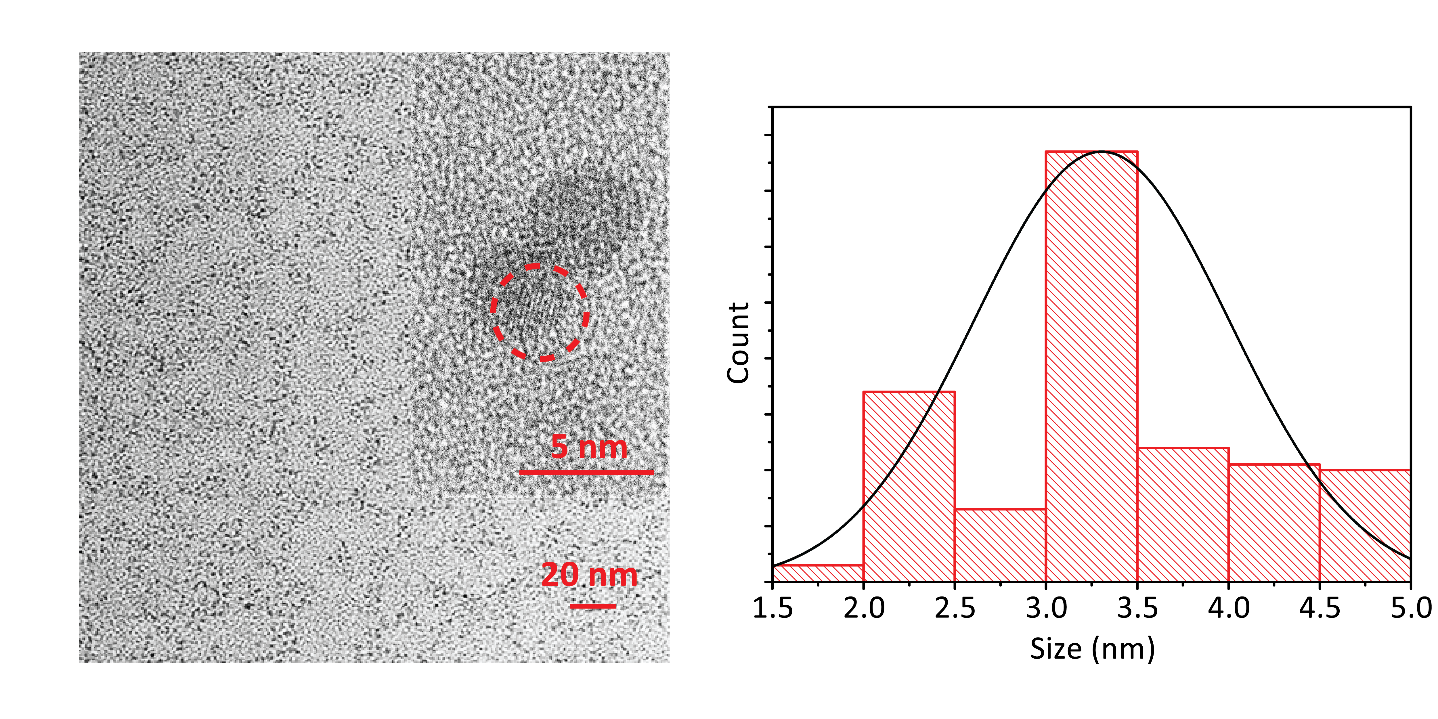


**Figure S1.** Transmission Electron Microscopy (TEM) image (inset: HR-TEM image) of InP core QDs and the corresponding size distribution (200 particles were counted).


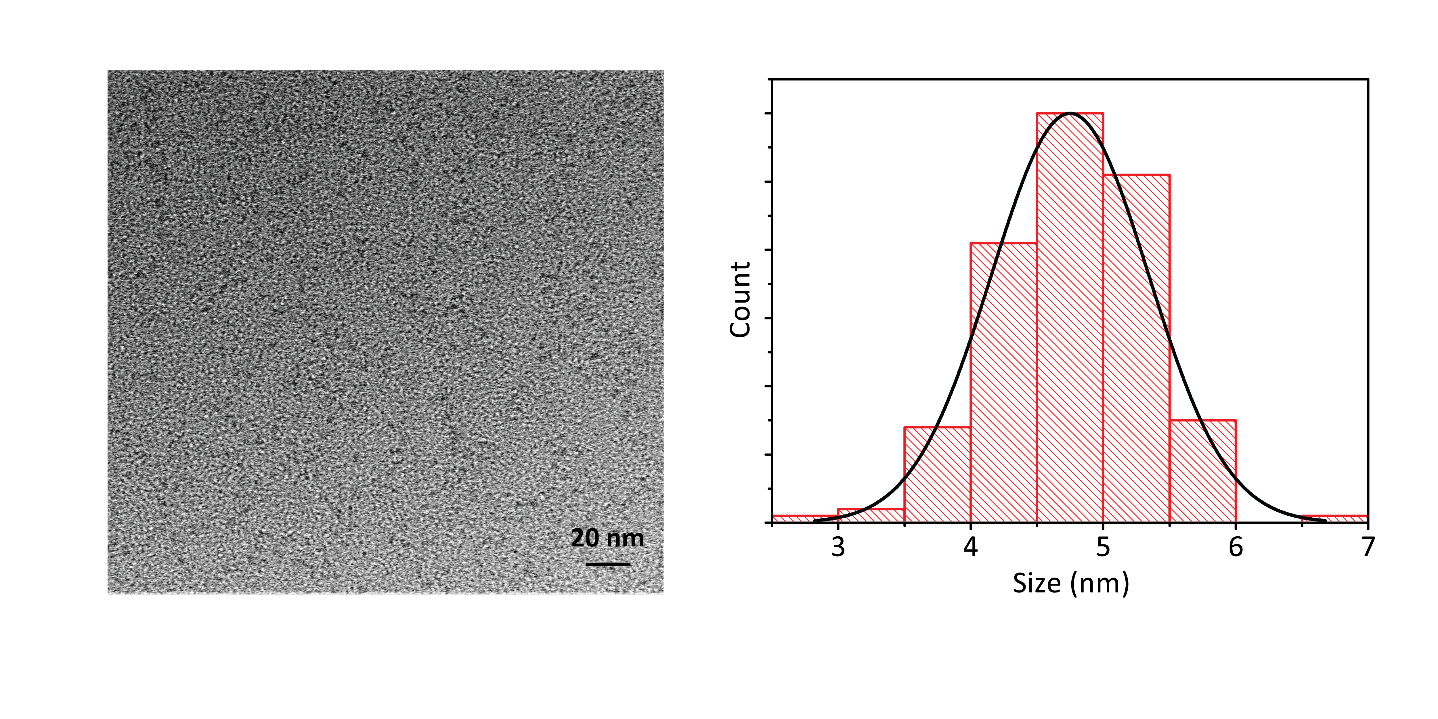


**Figure S2.** Transmission Electron Microscopy (TEM) image of InP/ZnS core/shell QDs and the corresponding size distribution (200 particles were counted).


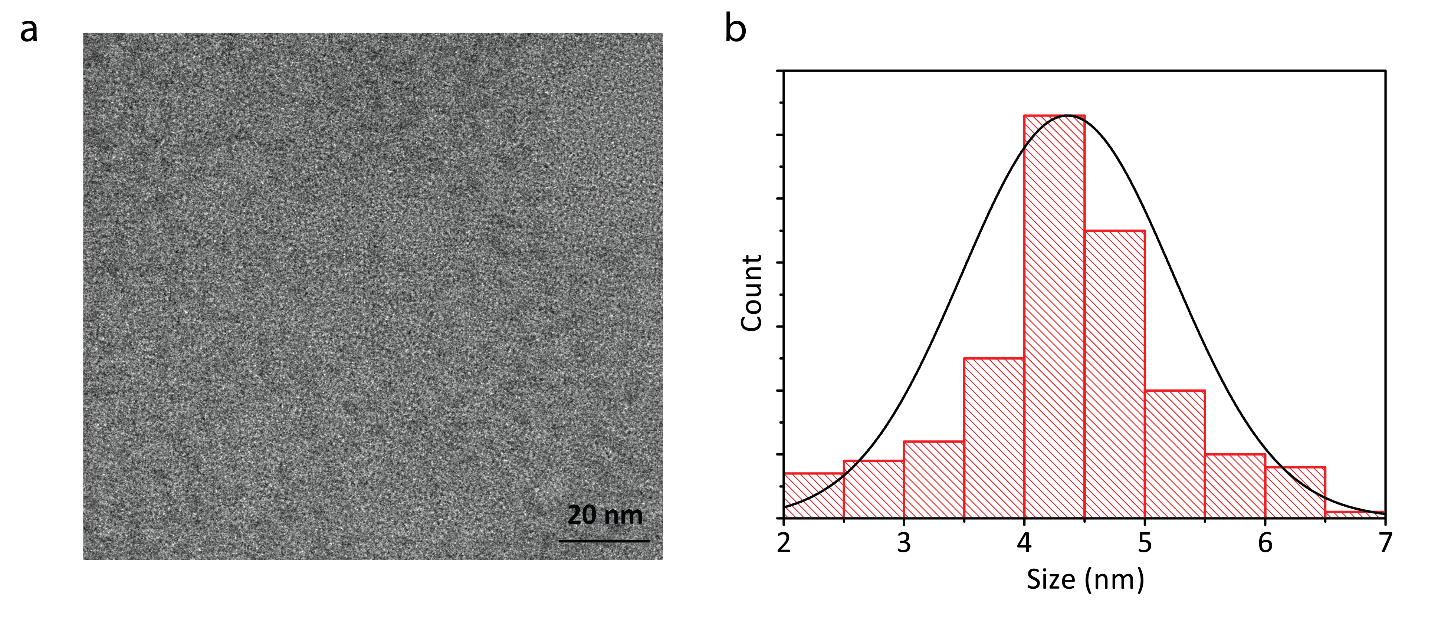


**Figure S3.** Transmission Electron Microscopy (TEM) image of InP/ZnO core/shell QDs, and the corresponding size distribution (200 particles were counted).





**Figure S4.** Normalized absorbance of InP core, InP/ZnS core/shell and InP/ZnO/ZnS core/shell/shell QDs.





**Figure S5.** Normalized absorbance of QD:PCBM blend with QD:PCBM volume ratios of 1:1, 1:3 and 1:7.


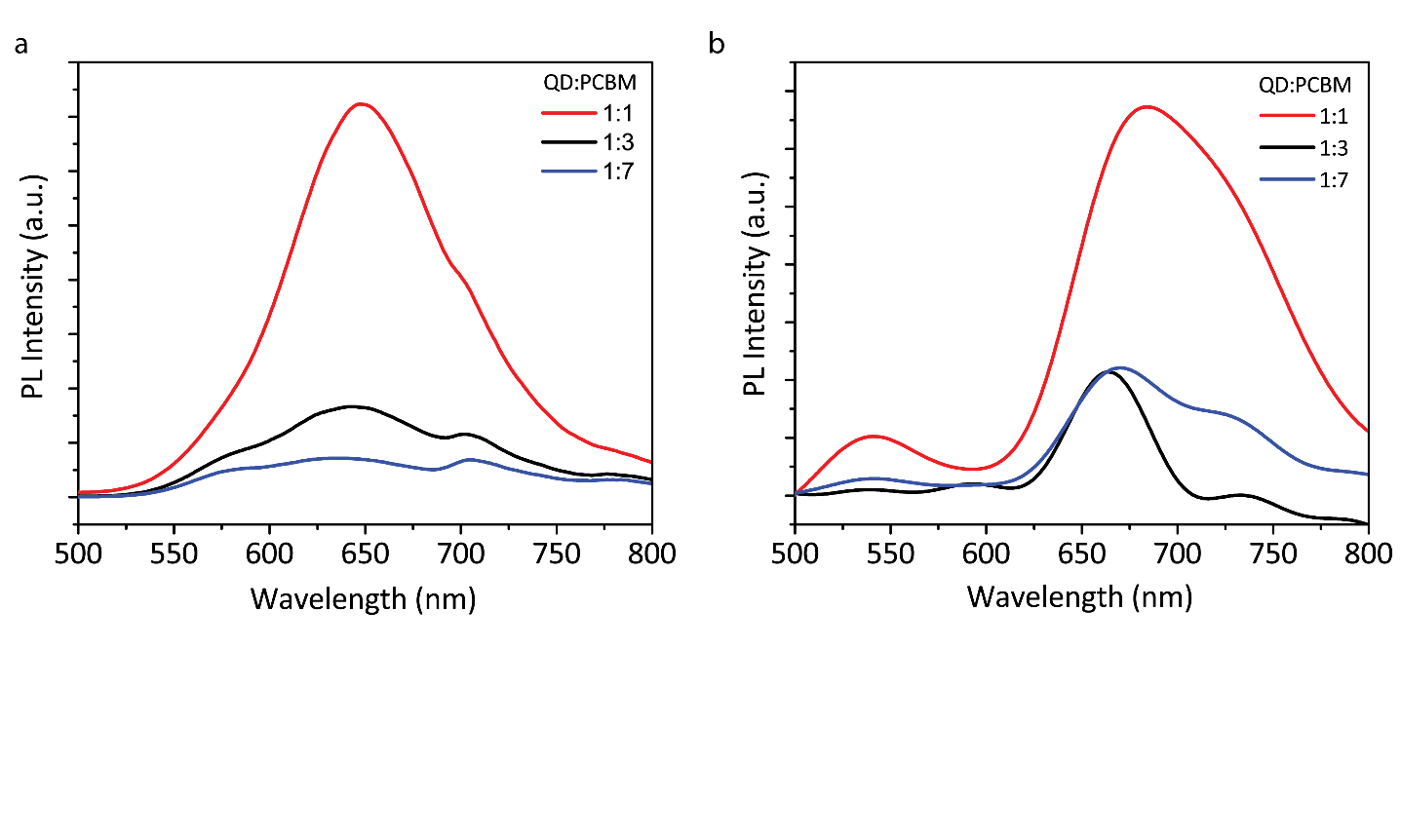


**Figure S6:** PL spectrum of a) QD:PCBM (1:1, 1:3, 1:7) mixture in solution (inset shows PL spectrum of PCBM) b) QD:PCBM mixture coated as thin film on the electrode.

**X-ray Photoelectron Spectroscopy (XPS) analysis of QDs**

Figure S7 shows the In 3d and P 2p XPS spectra of InP core QDs. In the In 3d spectrum (Figure S7a), the 444.4 eV and 452 eV peaks with a spin orbit splitting of 7.6 eV between them are characteristic of InP.^1-2^ P 2p spectrum of InP core QDs consists of two doublets, which denotes that P atoms participates in two distinct reactions (Figure S7b). The doublet in 132.4 eV – 133.3 eV range is associated to the P atoms in an oxidized medium, while the doublet in the 128.1 eV – 129 eV range is indicative of P^-3^ ions in InP.^3-4^ Besides, the 0.9 eV spin orbit splitting matches with the previous reports.^5^


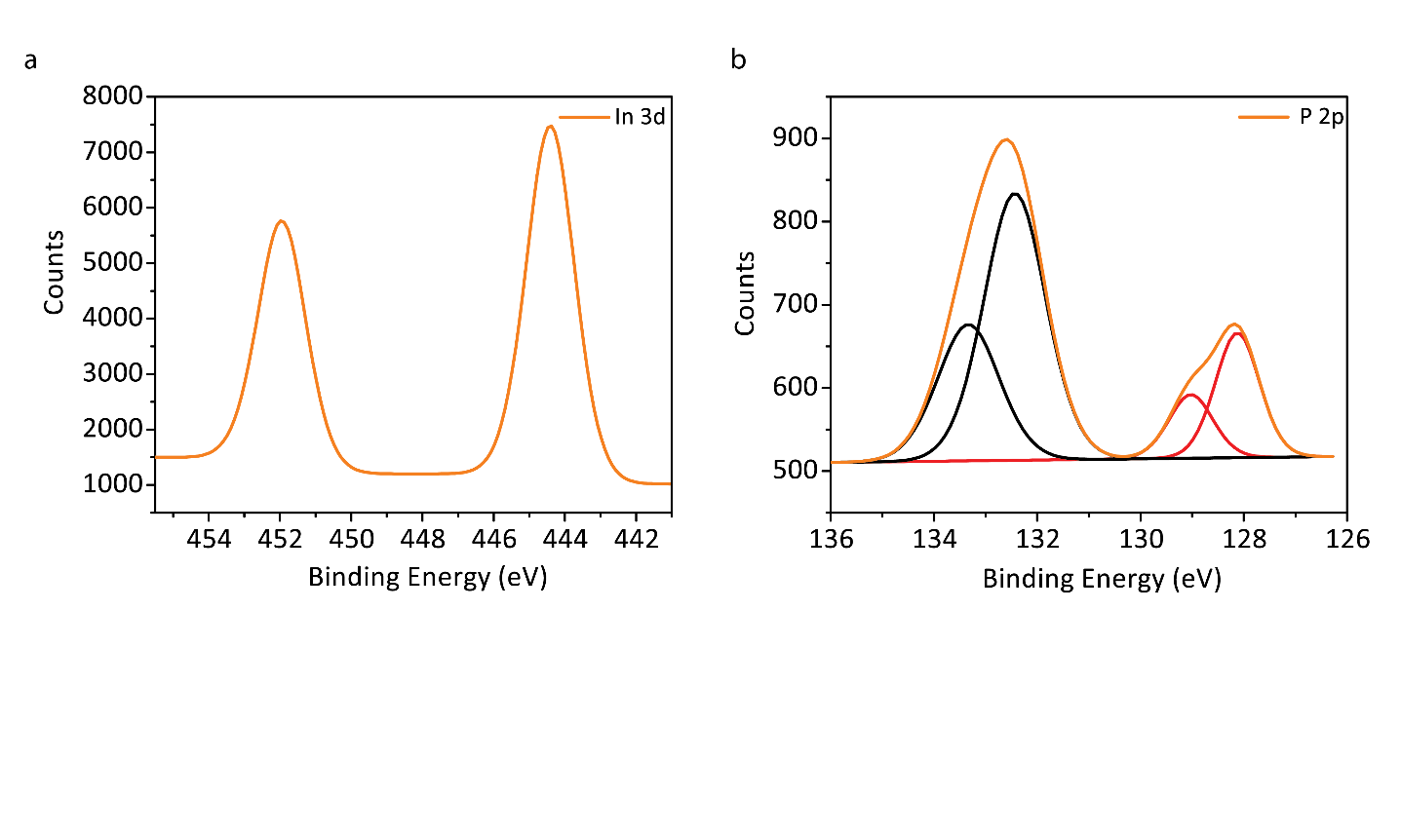


**Figure S7:** X-ray photoelectron spectroscopy analysis of InP core QDs. a) In 3d spectrum, b) P 2p spectrum.

For InP/ZnO core/shell QDs, we analyzed the Zn 2p and O 1s spectra to confirm ZnO shell growth (Figure S8). In the Zn-2p spectrum (Figure S8a), 2p_3/2_ and 2p_1/2_ peaks are observed at 1022.4 eV and 1045.4 eV, respectively, with 23 eV spin orbit splitting between them, which indicates the Zn^+2^ bound to oxygen in the ZnO.^6-7^ O 1s spectrum consists of a predominant peak at 531.6 eV, associated with lattice oxygen in the structure and a second peak at 532.8 eV (Figure S8b).^8-9^ Together, Zn 2p and O 1s spectra of InP/ZnO QDs suggest that ZnO shell is formed.


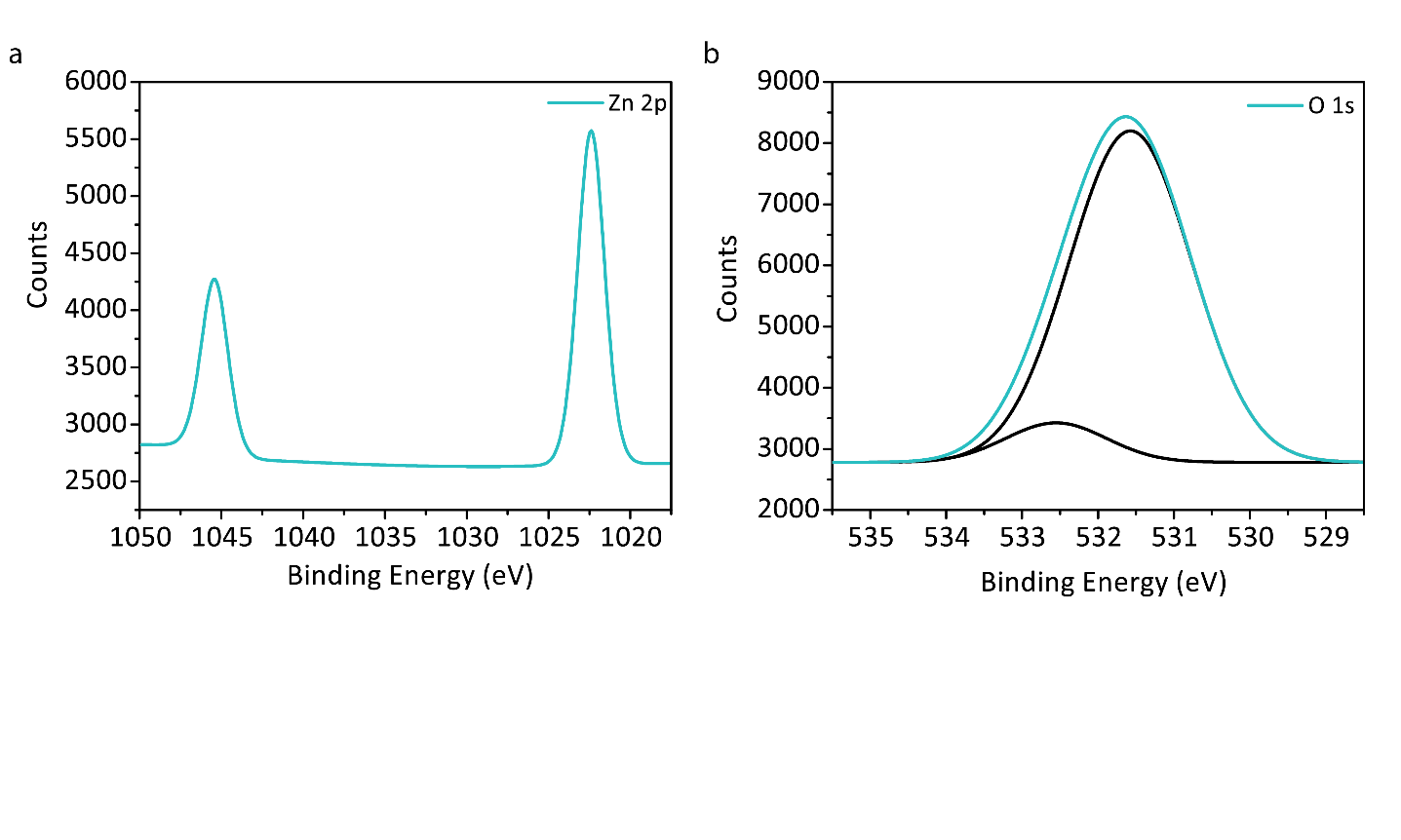


**Figure S8:** X-ray photoelectron spectroscopy analysis of InP/ZnO core/shell QDs. a) Zn 2p spectrum, b) O 1s spectrum.

Finally, to demonstrate the ZnS shell formation, we investigated the S 2p and Zn 2p XPS spectra of InP/ZnO/ZnS QDs (Figure S9). In the S 2p spectrum (Figure S9a), the asymmetric S 2p peak was deconvoluted into the subpeaks of S 2p_3/2_ and S 2p_1/2_ located at 161.1 eV and 162.4 eV, respectively, representing the S^-2^ anions in the ZnS_._^5^ The peak at 161.1 eV is the predominant one and associated with the S^-2^ in the ZnS structure.^5, 10^ Moreover, Zn 2p spectrum InP/ZnO/ZnS QDs exhibits Zn 2p_3/2_ and Zn 2p_1/2_ peaks at 1021.6 eV and 1044.6 eV, representing the Zn^+2^ ions (Figure S9b).^10^ The spin orbit splitting of 23 eV between Zn^+2^ peaks matches well with the previously reported values for Zn-S bond in the literature.^11-12^ Thus, the S 2p and Zn 2p spectra indicate the formation of ZnS shell.


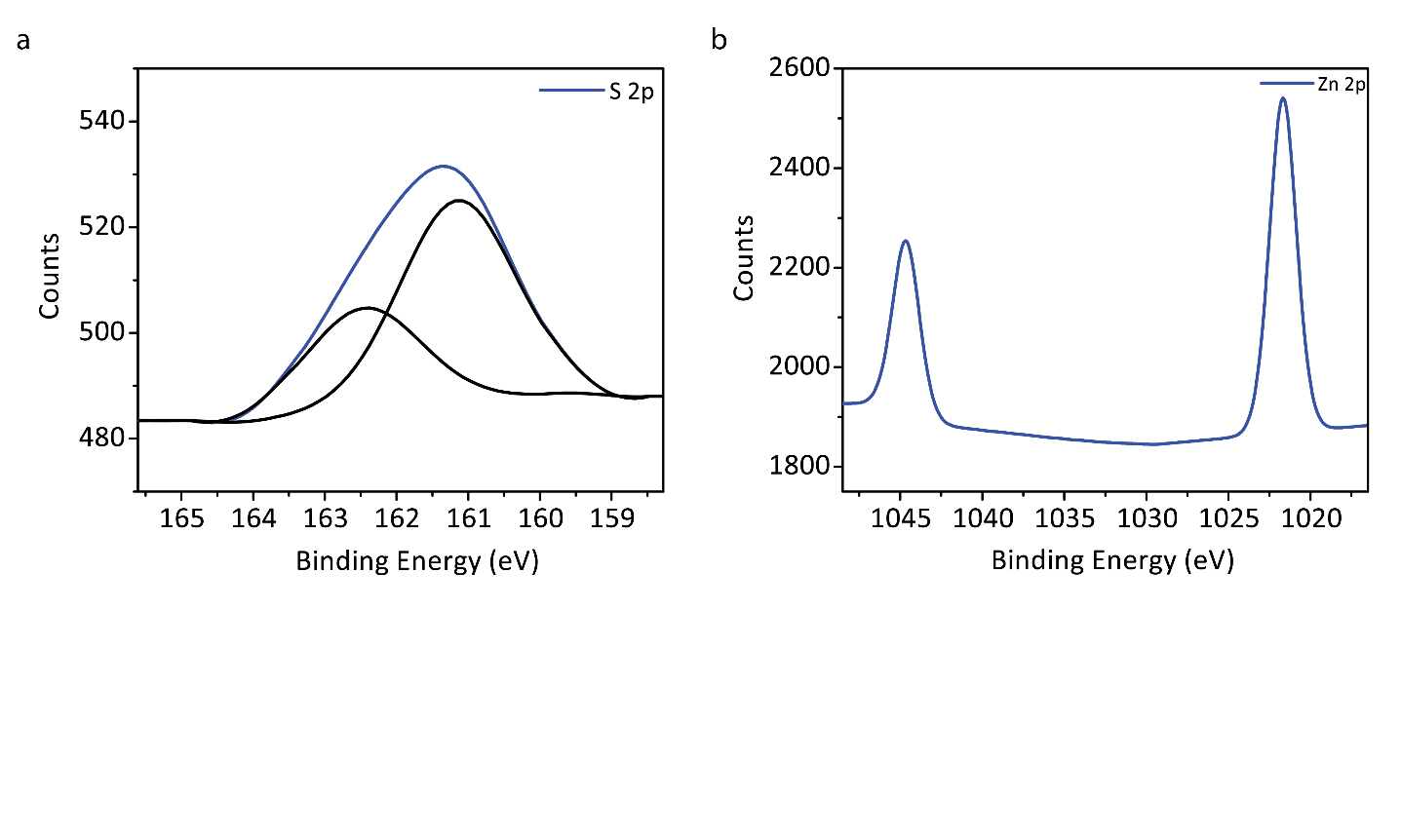


**Figure S9:** X-ray photoelectron spectroscopy analysis of InP/ZnO/ZnS core/shell/shell QDs. a) S 2p spectrum, b) Zn 2p spectrum.

**Energy dispersive X-ray spectroscopy (EDS) analysis of InP/ZnO/ZnS QDs**

We performed energy dispersive X-ray spectroscopy (EDS) of InP/ZnO/ZnS QDs for elemental mapping (Figure S10). Figure S10b shows the presence of In, P, Zn, S, O elements in the analyzed region of STEM image of InP/ZnO/ZnS QDs shown in Figure S10a. Existence of those elements supports the formation of InP/ZnO/ZnS core/shell/shell structure.


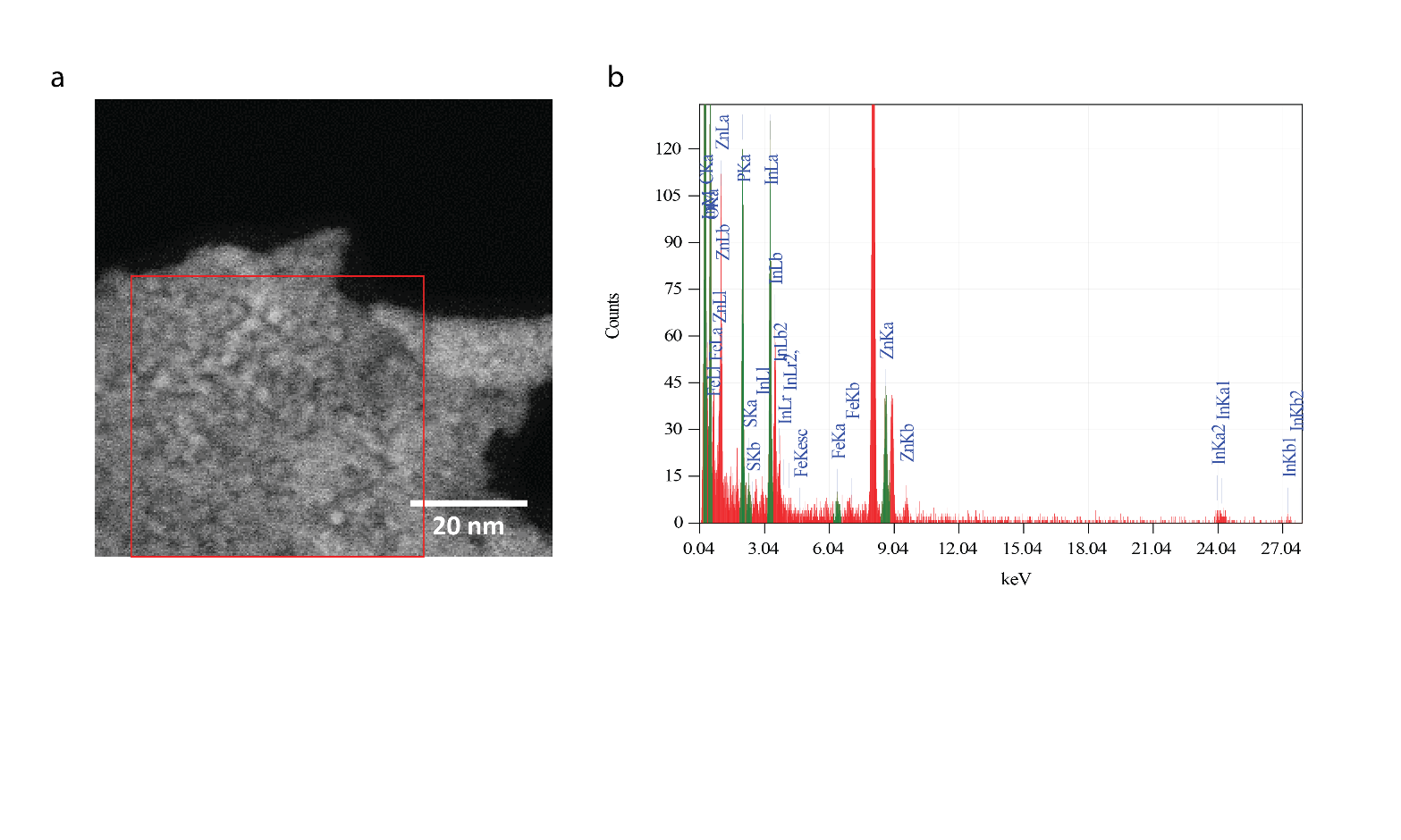

**Figure S10:** EDS elemental mapping of InP/ZnO/ZnS QDs. a) Z-contrast STEM image of QDs showing the areal region analyzed b) b) EDS spectrum of the region showing the presence of elements.


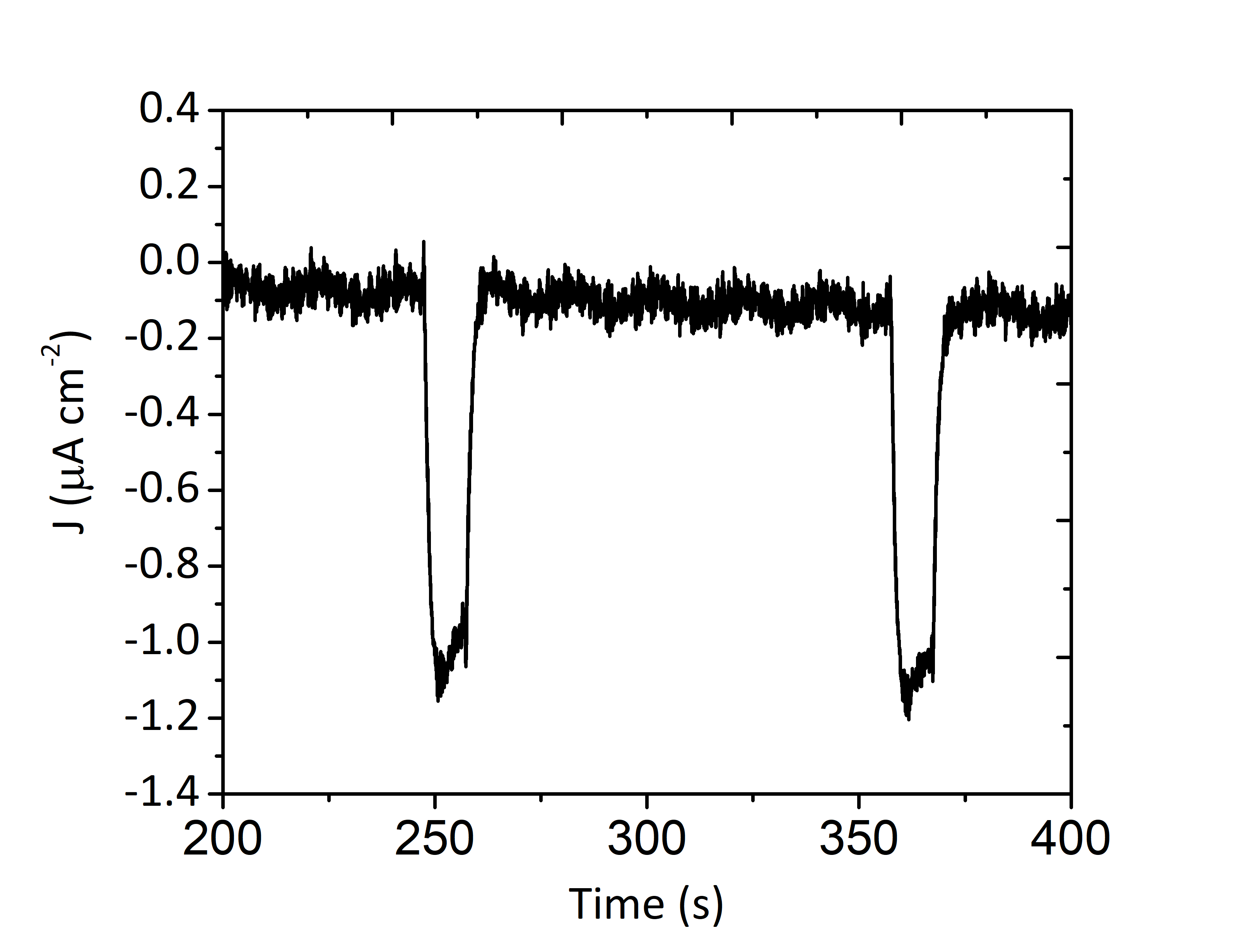


**Figure S11:** Photocurrent response of devices without QDs in ITO/ZnO/PCBM structure.

**Calculation of molar ratios of QD:PCBM and estimation of number of PCBM per QD**

The molar concentration of QD was estimated via Beer-Lambert law, which is formulized as:

$$A= \varepsilon cl (1)$$

where $A$ is the absorbance at the first excitonic peak, $\varepsilon$ is the extinction coefficient, $c$ is the molar concentration, $l$ is the path length, which is 1 cm due to the path length of the cuvette.

$\varepsilon$ can be calculated using the following empirical function:^13-14^

$$\varepsilon=3046.1\left( D^{3} \right)-76532\left( D^{2} \right)+\left( 5.5137*{10}^{5} \right)\left( D \right)-(8.9839*{10}^{5}) (2)$$

where $D$ is the diameter of the QD, which is 4.7 nm and 5.4 nm for InP/ZnS and InP/ZnO/ZnS, respectively.

For InP/ZnS, $\varepsilon$ = 318712.36 L mol^-1^ cm^-1^, $A$ = 0.14, $c$ = 0.45 $\mu$M

For InP/ZnO/ZnS, $\varepsilon$ = 326985.97 L mol^-1^ cm^-1^, $A$ = 0.16, $c$ = 0.49 $\mu$M

These concentrations are the ones used for absorbance measurements, which means they represent the diluted amounts (50 $\mu$l main QD solution in 3 ml toluene). Thus, the concentrations of main solutions are estimated as: c (InP/ZnS) = 26.4 $\mu$M, c (InP/ZnO/ZnS) = 29.4 $\mu$M

PCBM has molecular weight of 911 g mol^-1^. We use 30 mg ml^-1^ PCBM solution in the QD:PCBM blend, thus $c$ = 0.033 M.

We use three different QD:PCBM mixing ratios in the experiments: 1:1, 1:3, 1:7 volume ratios.

Hence, QD:PCBM molar ratios for each blend:

M_11_ = 0.08% for 1:1, M_13_ = 0.03% for 1:3, M_17_ = 0.01% for 1:7.

For InP/ZnO/ZnS:PCBM blend, we can calculate the number of PCBM per QD as following:

QD radius $r_{QD}$ = 2.7 nm and PCBM radius $r_{PCBM}$ = 0.55 nm.^15^

Assuming both QD and PCBM to be spheres, the geometry of QD:PCBM structure is schematized in Fig. S4. In that close-packed geometry, assuming a packing factor ($\eta$) of maximum 0.74, the number of PCBM per QD can be found by dividing the surface area of the outer circle to the cross-sectional area of PCBM and multiplying with the packing factor.

Thus, the maximum number of PCBM per QD is calculated as following:

$$N_{PCBM}=\frac{4\pi r_{out}^{2}}{\pi r_{PCBM}^{2}} \times\eta=103$$

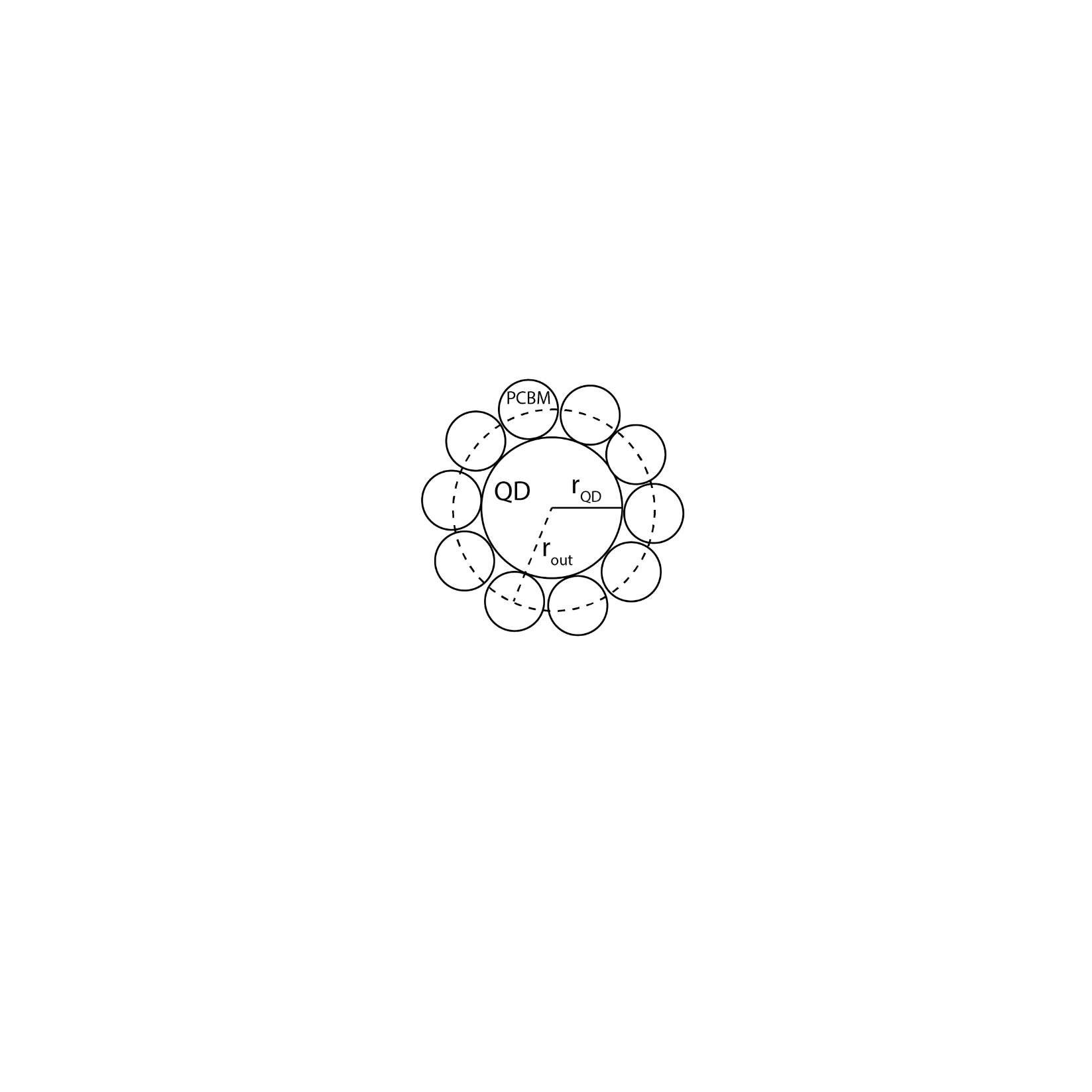


**Figure S11.** Schematic of QD:PCBM structure for calculation of number of PCBM per QD. QD and PCBM are assumed to be spherical. r_out_ is the radius of the outer circle shown with the dashed line, which is equal to the sum of the QD radius and PCBM radius.

**References**

1. Granada-Ramirez, D.; Arias-Cerón, J.; Gómez-Herrera, M.; Luna-Arias, J.; Pérez-González, M.; Tomás, S.; Rodríguez-Fragoso, P.; Mendoza-Alvarez, J., Effect of the indium myristate precursor concentration on the structural, optical, chemical surface, and electronic properties of InP quantum dots passivated with ZnS. *Journal of Materials Science: Materials in Electronics* **2019,** *30* (5), 4885-4894.

2. Ramasamy, P.; Kim, B.; Lee, M.-S.; Lee, J.-S., Beneficial effects of water in the colloidal synthesis of InP/ZnS core–shell quantum dots for optoelectronic applications. *Nanoscale* **2016,** *8* (39), 17159-17168.

3. Xi, L.; Cho, D.-Y.; Besmehn, A.; Duchamp, M.; Grützmacher, D.; Lam, Y. M.; Kardynał, B. E., Effect of zinc incorporation on the performance of red light emitting InP core nanocrystals. *Inorganic chemistry* **2016,** *55* (17), 8381-8386.

4. Granada-Ramirez, D.; Arias-Cerón, J.; Pérez-González, M.; Luna-Arias, J.; Cruz-Orea, A.; Rodríguez-Fragoso, P.; Herrera-Pérez, J.; Gómez-Herrera, M.; Tomás, S.; Vázquez-Hernández, F., Chemical synthesis and optical, structural, and surface characterization of InP-In2O3 quantum dots. *Applied surface science* **2020,** *530*, 147294.

5. Virieux, H.; Le Troedec, M.; Cros-Gagneux, A.; Ojo, W.-S.; Delpech, F.; Nayral, C. l.; Martinez, H.; Chaudret, B., InP/ZnS nanocrystals: coupling NMR and XPS for fine surface and interface description. *Journal of the American Chemical Society* **2012,** *134* (48), 19701-19708.

6. Moulder, J. F., Handbook of X-ray photoelectron spectroscopy. *Physical electronics* **1995**, 230-232.

7. Gao, D.; Zhang, Z.; Fu, J.; Xu, Y.; Qi, J.; Xue, D., Room temperature ferromagnetism of pure ZnO nanoparticles. *Journal of applied physics* **2009,** *105* (11), 113928.

8. Liu, L.; Chen, Y.; Guo, T.; Zhu, Y.; Su, Y.; Jia, C.; Wei, M.; Cheng, Y., Chemical conversion synthesis of ZnS shell on ZnO nanowire arrays: morphology evolution and its effect on dye-sensitized solar cell. *ACS applied materials & interfaces* **2012,** *4* (1), 17-23.

9. Ahmad, M.; Yan, X.; Zhu, J., Controlled synthesis, structural evolution, and photoluminescence properties of nanoscale one-dimensional hierarchical ZnO/ZnS heterostructures. *The Journal of Physical Chemistry C* **2011,** *115* (5), 1831-1837.

10. Liang, Y.-C.; Wang, C.-C., Surface crystal feature-dependent photoactivity of ZnO–ZnS composite rods via hydrothermal sulfidation. *RSC advances* **2018,** *8* (9), 5063-5070.

11. Wang, G.; Huang, B.; Li, Z.; Lou, Z.; Wang, Z.; Dai, Y.; Whangbo, M.-H., Synthesis and characterization of ZnS with controlled amount of S vacancies for photocatalytic H 2 production under visible light. *Scientific reports* **2015,** *5*, 8544.

12. Choi, Y. I.; Lee, S.; Kim, S. K.; Kim, Y.-I.; Cho, D. W.; Khan, M. M.; Sohn, Y., Fabrication of ZnO, ZnS, Ag-ZnS, and Au-ZnS microspheres for photocatalytic activities, CO oxidation and 2-hydroxyterephthalic acid synthesis. *Journal of Alloys and Compounds* **2016,** *675*, 46-56.

13. Reiss, P.; Protiere, M.; Li, L., Core/Shell semiconductor nanocrystals. *Small* **2009,** *5* (2), 154-68.

14. Devatha, G.; Roy, S.; Rao, A.; Mallick, A.; Basu, S.; Pillai, P. P., Electrostatically driven resonance energy transfer in “cationic” biocompatible indium phosphide quantum dots. *Chemical science* **2017,** *8* (5), 3879-3884.

15. Qiao, R.; Roberts, A. P.; Mount, A. S.; Klaine, S. J.; Ke, P. C., Translocation of C60 and its derivatives across a lipid bilayer. *Nano Letters* **2007,** *7* (3), 614-619.
